# A Speedy Point Cloud Registration Method Based on Region Feature Extraction in Intelligent Driving Scene

**DOI:** 10.3390/s23094505

**Published:** 2023-05-05

**Authors:** Deli Yan, Weiwang Wang, Shaohua Li, Pengyue Sun, Weiqi Duan, Sixuan Liu

**Affiliations:** 1 Hebei Provincial Collaborative Innovation Center of Transportation Power Grid Intelligent Integration Technology and Equipment, Shijiazhuang Tiedao University, Shijiazhuang 050043, China; yandl@stdu.edu.cn (D.Y.); lef1357@163.com (W.W.); 2 School of Electrical and Electronic Engineering, Shijiazhuang Tiedao University, Shijiazhuang 050043, China; pengyuesun25@gmail.com (P.S.); 18512413572@163.com (W.D.); liusixuan_echo@163.com (S.L.); 3 State Key Laboratory of Mechanical Behavior and System Safety of Traffic Engineering Structures, Shijiazhuang Tiedao University, Shijiazhuang 050043, China

**Keywords:** environment perception, cloud registration, feature extraction, rigid body change

## Abstract

The challenges of point cloud registration in intelligent vehicle driving lie in the large scale, complex distribution, high noise, and strong sparsity of lidar point cloud data. This paper proposes an efficient registration algorithm for large-scale outdoor road scenes by selecting the continuous distribution of key area laser point clouds as the registration point cloud. The algorithm extracts feature descriptions of the key point cloud and introduces local geometric features of the point cloud to complete rough and fine registration under constraints of key point clouds and point cloud features. The algorithm is verified through extensive experiments under multiple scenarios, with an average registration time of 0.5831 s and an average accuracy of 0.06996 m, showing significant improvement compared to other algorithms. The algorithm is also validated through real-vehicle experiments, demonstrating strong versatility, reliability, and efficiency. This research has the potential to improve environment perception capabilities of autonomous vehicles by solving the point cloud registration problem in large outdoor scenes.

## 1. Introduction

Accurately obtaining information about the environment around the vehicles is critical to the safety of autonomous vehicles [1]. Environment perception is a mechanism that provides a natural and dense understanding of the relationship between objects and their surroundings. It plays a critical role in enabling autonomous vehicles to operate safely and make informed decisions by classifying the importance of environmental factors and providing real-time and accurate information about the surroundings. Lidar is commonly used in self-driving cars to obtain real-time information about the vehicle’s surroundings [2], and 3D point cloud data is used to perceive the changes in the environment as the car moves. However, 3D point cloud data usually contain noise, lack obvious spatial topological relationships, and have the problem of repeatedly scanning an object many times. In view of the above problems, current researchers solve them through point cloud registration technology. Point cloud registration is a fundamental problem in computer vision and plays a vital role in autonomous driving [3].

The Iterative Closest Point (ICP) algorithm [4] is a widely used point cloud registration technique. It works by establishing a distance threshold between two sets of point cloud data, identifying corresponding points between them, and iteratively solving for the optimal transformation matrix between the two sets. However, the accuracy and efficiency of the algorithm can be significantly affected by the initial position of the point cloud. In response to this problem, some scholars have proposed several point cloud registration algorithms that combine ICP and different coarse registration algorithms [5,6,7]. To achieve the optimal estimation of the global point cloud, some scholars have proposed several different versions based on ICP [8,9,10]. In addition, some scholars proposed to use the neighborhood feature descriptor of the point cloud to accelerate point cloud registration [11,12,13]. This method only uses a small number of point clouds for point cloud registration, which can shorten the registration time of point clouds, but the registration accuracy of point clouds cannot be guaranteed. As deep learning continues to evolve, several point cloud registration algorithms based on deep learning have emerged [14,15,16,17]. However, these algorithms are currently limited to object-level or indoor point clouds and may not be suitable for large-scale outdoor autonomous-driving point clouds.

To improve the registration accuracy of lidar point clouds and overcome various challenges, several approaches have been proposed in the recent literature. NRLI-UAV, proposed in [18], addresses the issue of registration failure when rigid transformations are used between images and lidar point clouds. In [19], the global registration of subway- tunnel point clouds is studied by using an enhanced extended Kalman filter and central axis constraints; [20] proposes a novel solution for point cloud registration in areas with low overlap. The data acquired by the oblique airborne photogrammetry system AOS-Tx8 are processed using a new processing scheme developed in [21], which aims to create large-scale regional thermal property maps. A 3D global localization method for identifying the location of objects in underground mines is proposed in [22]. Using mobile lidar mapping and point cloud registration, [23] proposes an adaptive feature region extraction method for simplifying point cloud data and improving registration accuracy. Ref. [24] proposes a registration algorithm that integrates road information constraints to improve the registration accuracy of Lidar-Inertial measurement unit (Imu) odometry in urban environments. Ref. [25] proposes a Lidar-Imu-Global Navigation Satellite System (GNSS) fusion positioning algorithm based on voxelized precise registration to address the problem of insufficient registration accuracy and cumulative errors. Ref. [26] proposes a pipeline internal localization method based on data fusion and point cloud registration to improve the positioning accuracy of pipeline robots. Ref. [27] proposes a symmetry-based lidar point registration method, which derives 3-D central axes from multi-source point clouds using object symmetry.

Intelligent-vehicle lidar point clouds have unique characteristics, such as high sparsity, large spatial extent, and complex and variable distributions [28], which make traditional point cloud registration algorithms inadequate. Many existing algorithms have the problems of slow convergence, long processing time, and single-application scenarios, making them unsuitable for intelligent-vehicle point cloud registration. This paper proposes a point cloud registration algorithm under the constraints of key point clouds and point cloud features, which solves the key problems of positioning or mileage calculation methods in intelligent driving and promotes the development of point cloud object detection and recognition. 

In summary, our main contributions are as follows:

(1) A new point cloud registration algorithm is proposed in this paper, which exhibits high accuracy, real-time performance, and reliability.
(2) The selection of point clouds in key regions makes only a very small number of point clouds available for registration.
(3) The local geometric features of the point cloud are introduced in our method to complete the point cloud registration process under the constraints of the key point cloud.

This research paper is structured as follows: Section 2 presents the point cloud registration algorithm proposed in this paper. In Section 3, the algorithm for the point cloud coarse registration phase is described. Section 4 elaborates the algorithm for the point cloud fine registration. Experimental validation is shown in Section 5. The conclusion of this research paper is given in Section 6.

## 2. Research Methods

In this section, the general framework of the algorithm and the method for continuous point cloud region extraction are introduced.

### 2.1. Algorithm Framework

The objective of point cloud registration is to determine the rigid body transformation matrix between two point clouds, such that the spatial distance between the transformed point cloud and the target point cloud is minimized. The mathematical language is expressed as follows [29]:

(1) $$T=\arg\min\sum_{i=1}^{n}{\left(P_{i}-T⊗P_{i}\right)}^{Τ}•(Q_{i}-T⊗Q_{i})$$

**Figure 1:**
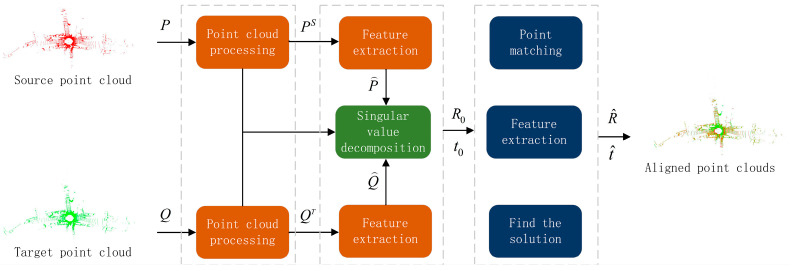
The algorithm framework.

In Equation (1), T is the rigid body transformation matrix between the two sets of point clouds, and $P_{i}$ and $Q_{i}$$\left(i=1,2,3\ldots n\right)$ are the n pairs of matching points in the two sets of point clouds. The overall framework of the point cloud registration algorithm proposed in this paper is shown in Figure 1.

Given source and target point clouds P, Q$\in {\mathbb{R}}^{N\times 3}$, the key point clouds $P^{S}=\{P_{1}^{S},P_{2}^{S},P_{3}^{S}\ldots P_{i}^{S}\}$ and $Q^{T}=\{Q_{1}^{T},Q_{2}^{T},Q_{3}^{T}\ldots Q_{i}^{T}\}$ are obtained by using the continuous key point cloud selection module of this paper. Coarsely registered point clouds $\overset{⌢}{P}=\{{\overset{⌢}{P}}_{1},{\overset{⌢}{P}}_{2},{\overset{⌢}{P}}_{3}\ldots {\overset{⌢}{P}}_{a}\}$ and $\overset{⌢}{Q}=\{{\overset{⌢}{Q}}_{1},{\overset{⌢}{Q}}_{2},{\overset{⌢}{Q}}_{3}\ldots {\overset{⌢}{Q}}_{a}\}$ are obtained under the Fast Point Feature Histogram (FPFH) feature constraint [30], which is used to solve the initial rotation matrix $R_{0}$ and initial translation vector $t_{0}$. After coarse registration, the relative positional relationship between point clouds is improved. Based on the evaluation of the point cloud normal vector and curvature, the optimal transformation matrix $\overset{⌢}{T}=\left[\begin{array}{cc} \overset{⌢}{R} & \overset{⌢}{t} \end{array}\right]$ is solved iteratively. $\hat{R}$ represents the rotation matrix, and $\hat{t}$ represents the translation vector.

### 2.2. Region of Interest Area

**Figure 2:**
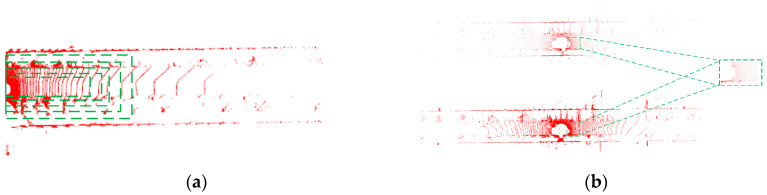
The algorithm framework: (**a**) Region of Interest region selection; (**b**) point cloud data preprocessing.

The pre-processed point cloud data in Figure 2 appears to be cleaner and more organized compared to the raw point cloud data, which may contain more noise and invalid points. The pre-processing step helps to remove these unwanted points and improve the quality of the point cloud data, which can lead to more accurate point cloud registration results.

**Figure 3:**
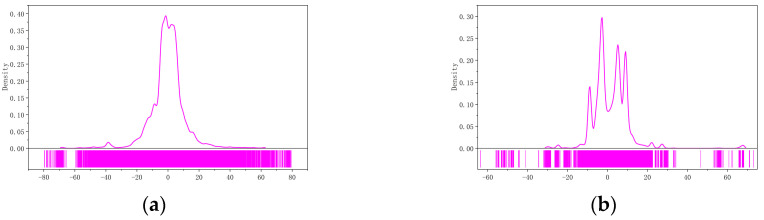
Vertical and horizontal distribution of point clouds: (**a**) x distribution; (**b**) y distribution.

It should be noted that the distribution of point clouds in intelligent driving scenarios is complex and changeable, and there is no obvious spatial topological relationship between point clouds. Figure 3 shows the vertical and horizontal distribution of point clouds in such scenes. To avoid focusing on a single scene, experiments on the KITTI dataset [31] (dataset for research in the field of autonomous driving jointly sponsored by Karlsruhe Institute of Technology and Toyota Technological University Chicago) have shown that point clouds are mainly distributed in the regions of (−26.7083,20.5833) m in the x direction and (−20.2343,23.9661) m in the y direction. Therefore, the selected Region of Interest (ROI) for point cloud registration should be within this range.

**Figure 4:**
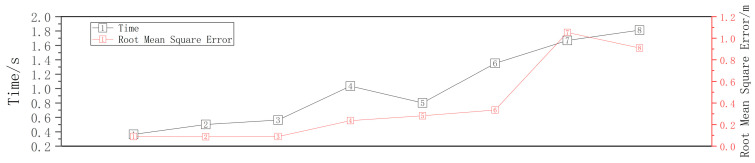
Time and root mean square error values for different ROI sizes.

In this study, different ROI regions are delineated by adjusting the vertical and horizontal thresholds of the point cloud. Figure 4 shows the impact of different ROI regions on the accuracy and efficiency of point cloud registration. Among them, the overall effect of ROI area 1 is ideal.

## 3. Point Cloud Coarse Registration

In this section, point clouds are characterized by FPFH features, and the initial change matrix $T_{0}$ between two frames of point clouds is computed.

### 3.1. FPFH Feature Descriptor

**Figure 5:**
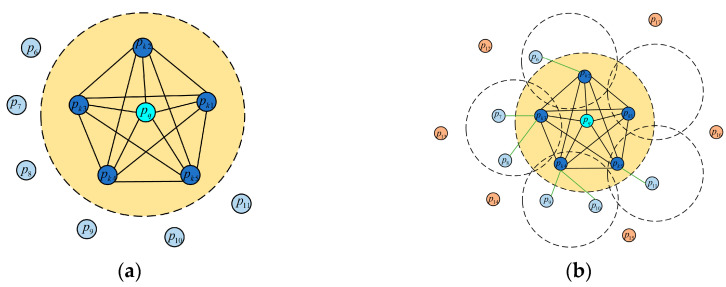
Comparison of influence areas: (**a**) PFH influence area; (**b**) FPFH influence area.

Different from the Point Feature Histogram (PFH) [32], the FPFH reduces the computational complexity from $O\left(nk^{2}\right)$ to $O\left(nk\right)$ by weighting the Simple Point Feature Histogram (SPFH). n represents the number of calculation points, and k represents the number of points within the radius of the calculation point field. Figure 5 is a comparison of the affected areas of the PFH and the FPFH.

For every pair of points $p_{i}$ and $p_{j}$$\left(i\neq j\right)$ in the k-neighborhood of each point p and their estimated normal $n_{i}$ and $n_{j}$, we compute the angular variations of $n_{i}$ and $n_{j}$ as follows:

(2) $$\alpha =\left(p_{j}-p_{i}\right)\times n_{i}•n_{j}$$

(3) $$\phi =\left(n_{i}•\left(p_{j}-p_{i}\right)\right)/\left\|p_{j}-p_{i}\right\|$$

(4) $$\theta =\arctan\left(n_{i}\times \left(p_{j}-p_{i}\right)\times n_{i}•n_{j},n_{i}•n_{j}\right)$$

In Equations (2)–(4), $n_{i}$ and $n_{j}$ are the estimated normal vectors of points $p_{i}$ and $p_{j}$, $\theta \in \left(0,2\pi \right)$. For each query point p, we compute the relationships (as shown in Equations (2)–(4)) between itself and its nearest neighbors, which is referred to as the “SPFH” calculation. For every point, we re-determine its nearest neighbors and utilize the SPFH values from k neighbors to weight the final calculation of the point’s feature descriptor, known as the “FPFH” descriptor:

(5) $$FPFH\left(p\right)=SPFH\left(p\right)+\frac{1}{k}\sum_{i=1}^{k}\frac{1}{\omega_{k}}SPFH\left(p_{k}\right)$$

In Equation (5), $\omega_{k}$ represents the distance between query point p and a neighbor point $p_{k}$ in a given metric space.

### 3.2. Singular Value Decomposition to Solve the Transformation Matrix

Singular Value Decomposition (SVD) [33] is a commonly used method for solving the rigid transformation between two point clouds by minimizing the least squares error. By computing the covariance matrix of the two point clouds, SVD can extract the rotation matrix and translation vector that align the two point clouds in the same coordinate system.

For the matched point sets $\overset{⌢}{P}=\{{\overset{⌢}{P}}_{1},{\overset{⌢}{P}}_{2},{\overset{⌢}{P}}_{3}\ldots {\overset{⌢}{P}}_{a}\}$ and $\overset{⌢}{Q}=\{{\overset{⌢}{Q}}_{1},{\overset{⌢}{Q}}_{2},{\overset{⌢}{Q}}_{3}\ldots {\overset{⌢}{Q}}_{a}\}$, the initial change matrix $T_{0}=\left[\begin{array}{cc} R_{0} & t_{0} \end{array}\right]$ is solved to minimize the spatial distance of the registered corresponding points, that is, to find the minimum error function:

(6) $$E\left(\begin{array}{cc} R_{0} & t_{0} \end{array}\right)=\sum_{i=1}^{a}{\left\|{\overset{⌢}{Q}}_{i}-R_{0}{\overset{⌢}{P}}_{i}-t_{0}\right\|}^{2}$$

In Equation (6), ${\overset{⌢}{Q}}_{i}$ and ${\overset{⌢}{P}}_{i}$ represent the points in the matched point sets $\overset{⌢}{P}$ and $\overset{⌢}{Q}$. To find the rotation matrix $R_{0}$, we change Equation (6) as follows:

(7) $$E\left(R_{0}\right)=\sum_{i=1}^{a}\left({\left({\overset{⌢}{Q}}_{i}-{\overset{⌢}{Q}}_{0}\right)}^{Τ}\left({\overset{⌢}{Q}}_{i}-{\overset{⌢}{Q}}_{0}\right)+{\left({\overset{⌢}{P}}_{i}-{\overset{⌢}{P}}_{0}\right)}^{Τ}\left({\overset{⌢}{P}}_{i}-{\overset{⌢}{P}}_{0}\right)-2{\left({\overset{⌢}{Q}}_{i}-{\overset{⌢}{Q}}_{0}\right)}^{Τ}R_{0}\left({\overset{⌢}{P}}_{i}-{\overset{⌢}{P}}_{0}\right)\right)$$

In Equation (7), ${\overset{⌢}{Q}}_{0}$ and ${\overset{⌢}{P}}_{0}$ are the centroids of point sets $\overset{⌢}{Q}$ and $\overset{⌢}{P}$. To minimize $E\left(R_{0}\right)$, the calculation needs to take the derivative of $E\left(R_{0}\right)$ and maximize $dE\left(R_{0}\right)$.

(8) $$dE\left(R_{0}\right)=tr\left(R_{0}\left({\overset{⌢}{P}}_{i}-{\overset{⌢}{P}}_{0}\right){\left({\overset{⌢}{Q}}_{i}-{\overset{⌢}{Q}}_{0}\right)}^{Τ}\right)$$

In Equation (8), $\left({\overset{⌢}{P}}_{i}-{\overset{⌢}{P}}_{0}\right){\left({\overset{⌢}{Q}}_{i}-{\overset{⌢}{Q}}_{0}\right)}^{Τ}$ is a third-order square matrix. In Equation (9), we denote $\left({\overset{⌢}{P}}_{i}-{\overset{⌢}{P}}_{0}\right){\left({\overset{⌢}{Q}}_{i}-{\overset{⌢}{Q}}_{0}\right)}^{Τ}$ by H.

(9) $$H=U\left[\begin{array}{ccc} \lambda_{1} & 0 & 0\\ 0 & \lambda_{2} & 0\\ 0 & 0 & \lambda_{3} \end{array}\right]V^{Τ}$$

In Equation (9), U and V are orthogonal matrices. $\lambda_{1}$, $\lambda_{2}$, $\lambda_{3}$ are the eigenvalues of the matrix H. The rotation matrix $R_{0}$ can be obtained by the following formula:

(10) $$R_{0}=VU^{T}$$

According to the obtained rotation matrix $R_{0}$, the translation vector $t_{0}$ is obtained by the following formula:

(11) $$t_{0}={\overset{⌢}{Q}}_{0}-R_{0}\frac{1}{a}\sum_{i=1}^{a}{\overset{⌢}{P}}_{i}$$

## 4. Point Cloud Fine Registration

In this section, local features of the point cloud are introduced, and the transformation matrix between matched point pairs is computed.

### 4.1. Extracting Point Cloud Features

**Figure 6:**
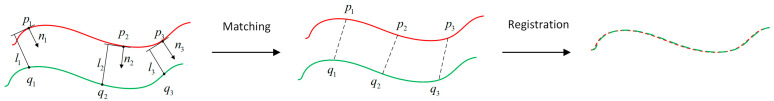
The principle of NICP. The red line represents the source point cloud, and the green line represents the target point cloud.

Different from ICP, Normal Iterative Closest Point (NICP) [34] takes the local features of the point cloud into consideration when matching two frames of point clouds, and uses the Levenberg-Marquardt (LM) algorithm to iteratively solve the point cloud transformation matrix. Figure 6 shows the principle of NICP.

To solve for the normal vector, we compute the covariance matrix of the Gaussian distribution of all points within a sphere of radius R around the target point $p_{i}$. If the point cloud surfaces in the target point domain are well-defined, they can be approximated as a plane, and only one of the eigenvalues of the covariance is much smaller than the other two. The eigenvector corresponding to the smallest eigenvalue is considered to be the normal vector of the point cloud plane of this point domain.

(12) $$\mu_{i}^{s}=\frac{1}{\left|\nu_{i}\right|}\sum_{p_{j}\in v_{i}}p_{j}$$

(13) $$\sum {}_{i}^{s}=\frac{1}{\left|\nu_{i}\right|}\sum_{p_{j}\in v_{i}}{\left(p_{j}-\mu_{i}^{s}\right)}^{Τ}\left(p_{j}-\mu_{i}^{s}\right)$$

In Equation (12), $\mu_{i}^{s}$ represents the centroid of the point cloud, and $v_{i}$ represents a cluster of point clouds in the field of a sphere of radius R in the target point cloud $p_{i}$. In Equation (13), $\sum {}_{i}^{s}$ is the Gaussian distribution covariance matrix.

(14) $$\sum {}_{i}^{s}=X\left[\begin{array}{ccc} \omega_{1} & 0 & 0\\ 0 & \omega_{2} & 0\\ 0 & 0 & \omega_{3} \end{array}\right]Y^{Τ}$$

In Equation (14), X and Y denote an orthogonal matrix after decomposing the matrix $\sum {}_{i}^{s}$. $\omega_{1}$, $\omega_{2}$, $\omega_{3}$ are the eigenvalues of $\sum {}_{i}^{s}$ in ascending order.

### 4.2. Searching for Matching Point Pairs

The points of the two sets of point clouds are projected into the same depth map, and those points that fall on the same pixel and have consistent normal vectors and curvatures are considered as matching point pairs. If multiple point clouds fall on the same pixel, the closest point pair is chosen, and their normal vectors must point to the same point. These alternative matching point pairs will be filtered by the following conditions, and if at least one of the following conditions is met, the group of point pairs will be removed.

The distance between two points exceeds a threshold:

(15) $$\left\|p_{i}^{c}-\hat{T}⊕p_{j}^{r}\right\|>\varepsilon_{d}$$

The absolute value of the difference between the logarithms of curvature of two points exceeds a threshold:

(16) $$\left|\log\sigma_{i}^{c}-\log\sigma_{j}^{r}\right|>\varepsilon_{\sigma }$$

The angle between the normal vectors of two points exceeds a threshold:

(17) $$n_{i}^{c}•\hat{T}⊕n_{j}^{r}<\varepsilon_{n}$$

In Equation (15), $\varepsilon_{d}$ represents the distance threshold, $\hat{T}$ represents the projection matrix, and $p_{i}^{c}$ and $p_{j}^{r}$ represent two matched points. In Equation (16), $\varepsilon_{\sigma }$ represents the curvature threshold, and $\sigma_{i}^{c}$ and $\sigma_{j}^{r}$ represent the curvatures of the two matching points. In Equation (17), $\varepsilon_{n}$ represents the angle threshold, and $n_{i}^{c}$ and $n_{j}^{r}$ represent the normal vectors of the two matching points.

### 4.3. Calculating the Transformation Matrix

When given a pair of matching points and the current transformation $\hat{T}$, the error function $e_{ij}\left(\hat{T}\right)$ is a six-dimensional vector.

(18) $$e_{ij}\left(\hat{T}\right)=\left({\tilde{p}}_{i}^{c}-{\tilde{p}}_{j}^{r}\right)$$

In Equation (18), ${\tilde{p}}_{i}^{c}$ and ${\tilde{p}}_{j}^{r}$ represent two matching points with normal vectors. Thus, the objective function composed of all point pairs is expressed as follows:

(19) $$\sum {}_{c}e_{ij}{\left(\hat{T}\right)}^{Τ}{\tilde{\Omega }}_{ij}e_{ij}\left(\hat{T}\right),$$

In Equation (19), ${\tilde{\Omega }}_{ij}$ is a 6 × 6 information matrix. The NICP algorithm uses a local parameterization of incremental perturbations to minimize the objective function $\sum {}_{c}e_{ij}{\left(\hat{T}\right)}^{Τ}{\tilde{\Omega }}_{ij}e_{ij}\left(\hat{T}\right)$; the increment is expressed as $\Delta T={\left(\begin{array}{cccccc} \Delta t_{x} & \Delta t_{y} & \Delta t_{z} & \Delta q_{x} & \Delta q_{y} & \Delta q_{z} \end{array}\right)}^{Τ}$, which contains the translation vector Δt and the imaginary part Δq of the rotation unit quaternion. By using the damped Gauss-Newton algorithm in each iteration of the calculation:

(20) $$\left(H+\lambda I\right)\Delta T=b$$

In Equation (20), $H=\sum J_{ij}{}^{Τ}\tilde{\Omega }J_{ij}$ is the approximated Hessian matrix, $b=J_{ij}{}^{Τ}\tilde{\Omega }e_{ij}\left(\hat{T}\right)$. The increment ΔT can be calculated by Equation (20), and $\hat{T}$ is updated.

(21) $$\hat{T}\leftarrow \Delta T⊕\hat{T}$$

$J_{ij}$ represents the Jacobian calculation, which is defined as follows:

(22) $$J_{ij}=\frac{\delta e_{ij}\left(\Delta T⊕\hat{T}\right)}{\delta \Delta T}$$

## 5. Experiments

This section begins with the visual and experimental data analysis of the proposed algorithm under various working conditions and scenarios. The raw data for the experiment are obtained from two lidars with completely different metrological characteristics. The point cloud registration experiment is conducted on a computer hardware environment consisting of an Intel(R) Core™ i5-11400H CPU and 16 GB memory.

### 5.1. Object-Level Point Cloud Registration Experiment

Outdoor point clouds can be considered as being composed of object-level factor clouds. Therefore, firstly, we used the rabbit point cloud from the public point cloud library provided by Stanford University for registration experiments and compared it with different common point cloud registration algorithms such as Normal Distribution Transformation (NDT), Trimmed Iterative Closest Point (TRICP), and NICP. Table 1 indicates the experimental effects of distinct registration algorithms under the rabbit point cloud of Stanford University.

**Figure 7:**
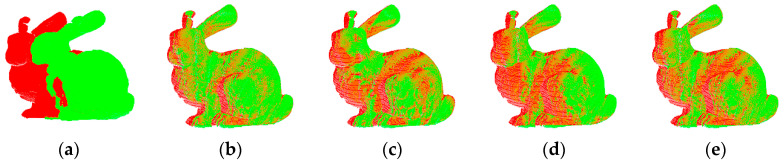
Stanford University Rabbit renderings. (**a**) Primitive point, (**b**) NDT, (**c**) TRICP, (**d**) NICP and (**e**) OURS.

The number of point clouds of the Stanford University rabbit model is about 40,000, which can be compressed to about 6000 after speedy sampling of the point cloud. From the consequences of rabbit registration at Stanford University in Table 1, it can be viewed that the running time of the algorithm proposed in this paper is 1.53 s. Compared with the different three registration algorithms, the time is significantly shortened, and the average registration time is shortened by 12.96 times. At the same time, the registration accuracy is slightly improved, and the average registration accuracy is increased by 11.56%. Figure 7 indicates the registration impact of different registration algorithms under the rabbit point cloud of Stanford University.

### 5.2. Multi-Condition Registration Experiment

**Figure 8:**
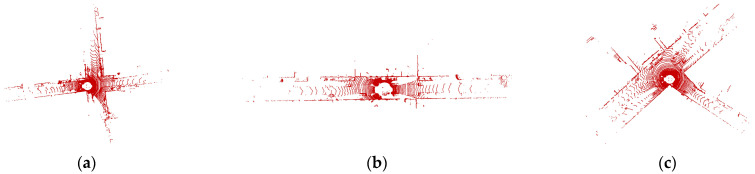
Different working conditions of point cloud registration. (**a**) Crossroads, (**b**) vehicle going straight, and (**c**) vehicle turning.

The KITTI dataset is a widely used benchmark dataset for evaluating algorithms related to autonomous driving, including point cloud registration. It contains data from a variety of urban, rural, and highway scenes with different levels of complexity, making it a good dataset for testing the robustness and accuracy of algorithms under different conditions. The Velodyne HDL-64E lidar used in the KITTI dataset is a high-performance sensor that can capture detailed and accurate point cloud information with a large coverage range, making it suitable for real-world autonomous driving applications. Figure 8 shows the point cloud distribution under different working conditions.

Table 2 shows the time registration results of different registration algorithms under the KITTI mileage dataset. In Table 2, scenarios 2, 3, and 6 are intersection conditions, scenarios 1, 4, and 8 are straight-going conditions, and scenarios 5, 7, and 9 are turning conditions.

**Figure 9:**
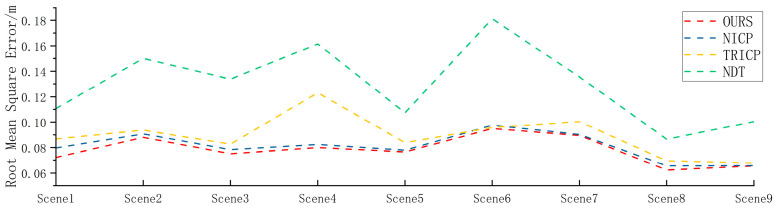
Accuracy comparison of multi-condition algorithms.

It is clear from Table 2 that the proposed algorithm has a significantly faster registration time compared to the other three algorithms, with an average registration time of 0.66 s. The NDT algorithm has the slowest registration time, with an average of 15.29 s, while the TRICP and NICP algorithms have registration times of 12.15 s and 14.00 s on average, respectively. In terms of registration accuracy, Figure 9 shows that the proposed algorithm also outperforms the other three algorithms, with an average root mean square error of 0.0787 m. On average, the proposed algorithm achieves an improvement of 17.75% in accuracy compared to the other three algorithms.

**Figure 10:**
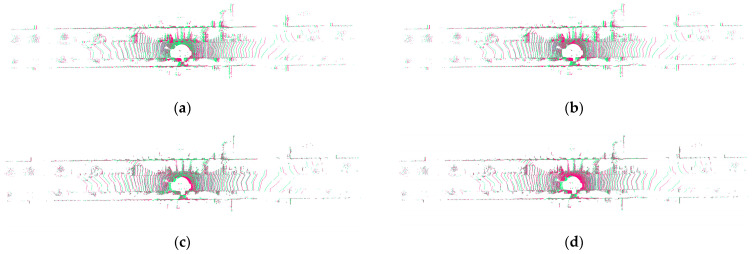

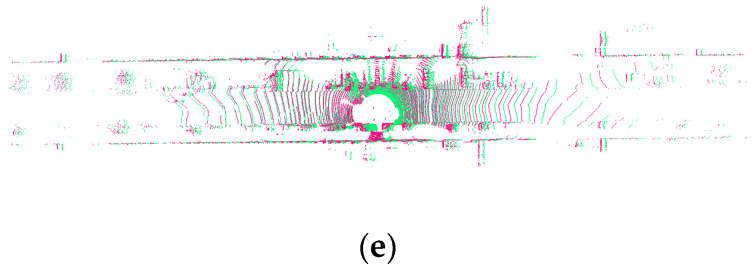
The registration effect of different algorithms under the straight driving condition in the street. (**a**) NDT, (**b**) NICP, (**c**) OURS, (**d**) TRICP, and (**e**) primitive point.

Figure 10 is a comparison of the effects of different point cloud registration algorithms under the KITTI mileage data. Among them, the registration effect of the NDT and NICP algorithms is not ideal, and the coincidence degree between the two frame point clouds after registration is not high. The TRICP registration effect is better, but the registration time is too long to meet the actual needs. It can be seen from Figure 10 that after the two frames of point clouds are registered by the algorithm of this paper, the ground, buildings, vehicles, and other objects have achieved a high degree of fusion. Moreover, the corresponding relationship between point clouds is very stable and will not change due to external interference. At the same time, the correspondence between point clouds is very complete, and there are no missing or repeated point clouds. The algorithm proposed in this paper has strong adaptability and can meet the needs of point cloud registration in different working conditions.

### 5.3. Multi-Scene Registration Experiment

**Figure 11:**
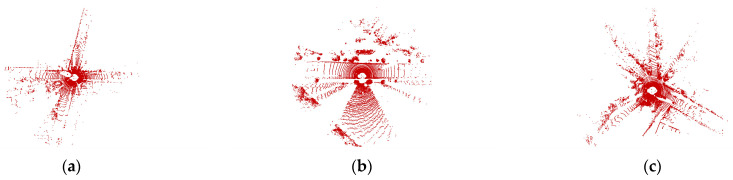
Different scenarios for point cloud registration. (**a**) City scene, (**b**) road scene, and (**c**) rural scene.

The distribution of point clouds in different scenarios is very different, which brings great challenges to point cloud registration. In order to verify the versatility of the algorithm proposed in this paper in different scenarios, several frames of data in urban, rural, and road scenes were selected from the KITTI dataset to conduct extensive verification experiments. Figure 11 is the distribution of point clouds in different scenarios.

**Figure 12:**
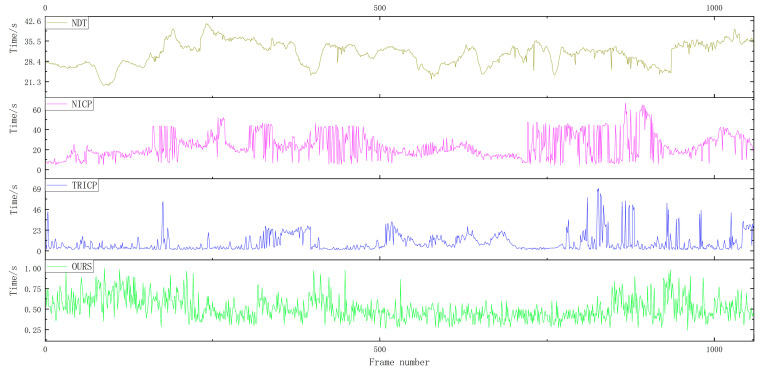
Time comparison of the four algorithms in the road scene.

For the verification experiments, a total of 1059 frames of road point cloud data were selected. Figure 12 shows the temporal analysis of point cloud registration for four different algorithms in road scenes. The average point cloud registration time for the proposed algorithm in this paper is 0.5084 s, compared to 8.7548 s for the TRICP algorithm, 22.3652 s for the NICP algorithm, and 31.0748 s for the NDT algorithm.

**Figure 13:**
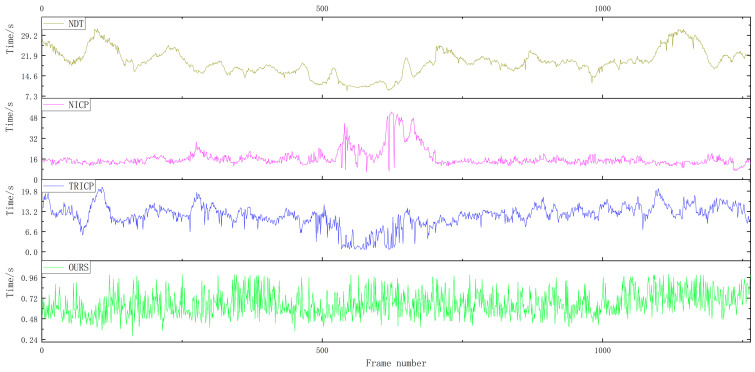
Time comparison of the four algorithms in the rural scene.

A total of 1264 frames of rural point cloud data were selected for verification experiments. Figure 13 shows the time analysis of four different algorithms for point cloud registration in rural scenes. The proposed algorithm in this paper has an average point cloud registration time of 0.6555 s, which is significantly shorter compared to the TRICP algorithm with an average point cloud registration time of 11.8145 s, the NICP algorithm with an average point cloud registration time of 16.5529 s, and the NDT algorithm with an average point cloud registration time of 19.4471 s.

**Figure 14:**
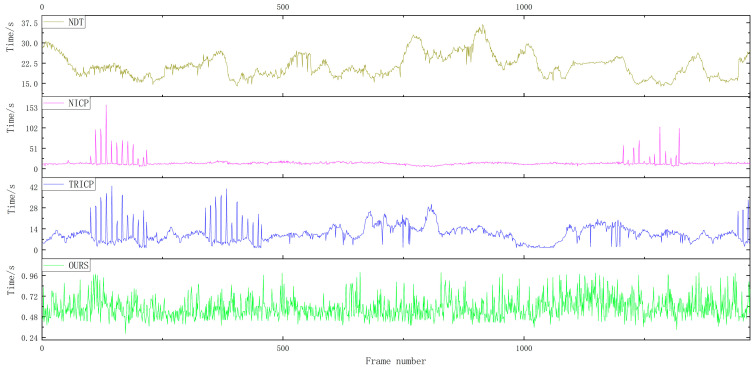
Time comparison of the four algorithms in the urban scene.

A total of 1469 frames of urban point cloud data were selected for verification experiments. Figure 14 shows the time analysis of four different algorithms for point cloud registration in urban scenes. For the verification experiments, 1469 frames of urban point cloud data were selected. The average point cloud registration time of the proposed algorithm was 0.5855 s, while the average registration times of the TRICP, NICP, and NDT algorithms were 10.7177, 14.0146, and 21.5313 s, respectively.

Among the four point cloud registration algorithms, NDT takes the longest time, and the registration efficiency varies greatly in different scenarios; the registration efficiency of NICP and TRICP algorithms is relatively stable, but the registration time in some scenarios is too long and the overall registration time cannot meet the real-time requirements. In the road, rural, and urban scenarios, the algorithm proposed in this paper maintains a stable calculation time, and the average registration time is 0.5831 s. Thanks to the selection of the ROI area of the point cloud in front of the vehicle, it avoids large-scale searching for the corresponding relationship between point clouds, which can greatly shorten the time for point cloud registration.

**Figure 15:**
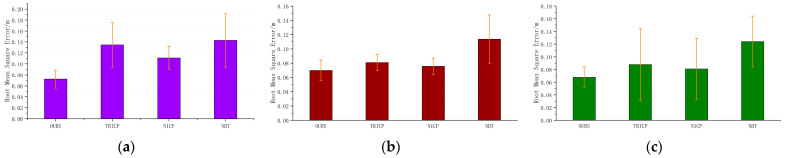
Accuracy comparison of four algorithms in different scenarios. (**a**) Road scene, (**b**)rural scene, and (**c**) urban scene.

Table 3 shows the accuracy comparison of various algorithms in different scenarios. The average registration value of the NDT algorithm is 0.1268 m, the average registration value of the NICP algorithm is 0.08910 m, and the average registration value of the TRICP algorithm is 0.1011 m. Among them, the registration accuracy of the NDT algorithm is stable, but the error is large and does not meet the actual needs; the TRICP and NICP algorithms maintain excellent performance in some scenarios, but the algorithms are not universal and cannot adapt to different scenarios. The average value of the algorithm proposed in this paper is 0.06996 m. In road, rural, and urban scenarios, the algorithm in this paper maintains high accuracy and stability. Figure 15 is a comparison of multiple algorithms in different scenarios.

### 5.4. Real-Vehicle Registration Experiment

To evaluate the effectiveness of the proposed point cloud registration algorithm, experiments were conducted using an electric vehicle with a Hesai 40-line lidar, a Changjiang No. 3 camera, and an inertial measurement unit, driven by the researchers at the State Key Laboratory. The sensors were rigidly connected to establish a stable coordinate system conversion relationship, and after data processing and coordinate system conversion, accurate three-dimensional space coordinates of each scanned object were obtained. Table 4 shows the lidar sensor parameters.

**Figure 16:**
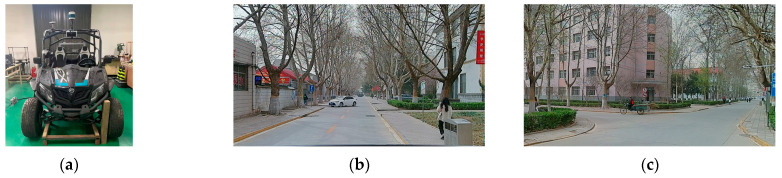
General situation of real-vehicle experiment. (**a**) Experimental platform, (**b**) straight driving condition, and (**c**) intersection condition.

The test area for the study was mainly located in the Shijiazhuang Railway University headquarters campus, which is composed of campus roads. The road conditions in this area are relatively flat, without any significant bumpy road sections. The data collection process involved collecting point clouds of various objects, including pedestrians, roads, trees, buildings, and speed bumps around the vehicle body. The collected data was very accurate, complete, and without any missing or repeated point clouds. This allowed for a complete reflection of the scene’s characteristics and details, facilitating comprehensive analysis and processing. The data coverage was extensive, covering every corner and detail of the entire scene. Figure 16 shows the equipment installation location and real-vehicle data collection environment.

**Figure 17:**
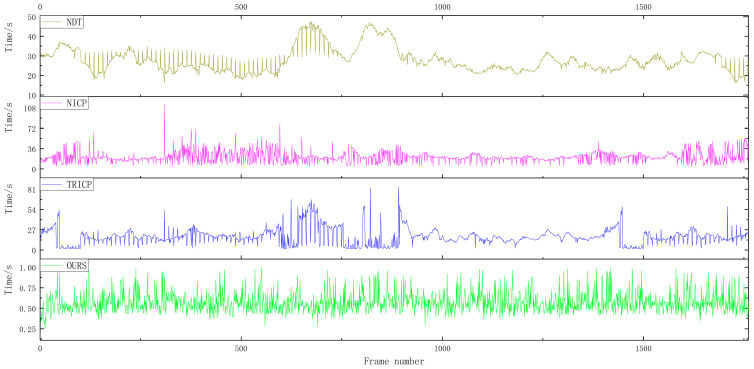
Analysis of registration time of real-vehicle point cloud.

A total of 1761 frames were selected from the collected point cloud data for verification experiments. Figure 17 shows the point cloud registration time analysis of the real vehicle. The average point cloud registration time of the algorithm proposed in this paper is 0.5734 s, the average point cloud registration time of the TRICP algorithm is 17.8066 s, the average point cloud registration time of the NICP algorithm is 21.1194 s, and the average point cloud registration time of the NDT algorithm is 27.8159 s. The time efficiency of the algorithm proposed in this paper still maintains a high real-time performance under the real-vehicle test. It can be seen from Figure 17 that the longest registration time of NDT is 47.3280 s, the longest registration time of NICP is 114.8751 s, and the longest registration time of TRICP is 82.8434 s. The algorithm in this paper maintains high stability, and there is no phenomenon where the registration time of a certain point cloud is too long.

**Figure 18:**
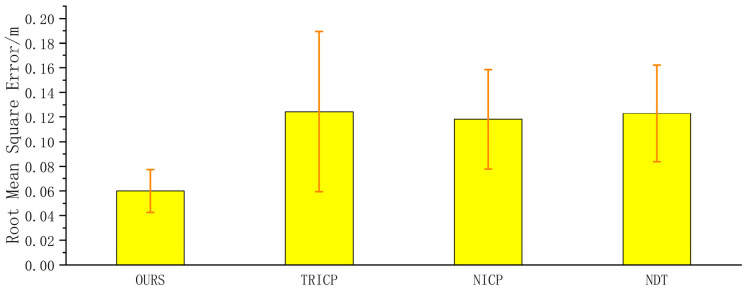
Accuracy analysis of point cloud registration in real vehicle.

Table 5 shows the accuracy analysis of various algorithms under real-vehicle experiments. Among them, the maximum error range of the registration accuracy of the TRICP algorithm is 0.06505 m, followed by NICP and NDT. The minimum error range of the algorithm proposed in this paper is 0.01751 m. At the same time, compared with the other three algorithms, the algorithm proposed in this paper has the highest registration accuracy. Experimental results demonstrate the versatility and high accuracy of the registration algorithm proposed in this paper under real-vehicle conditions. Figure 18 is the analysis of the registration accuracy of the real-vehicle point cloud.

**Figure 19:**
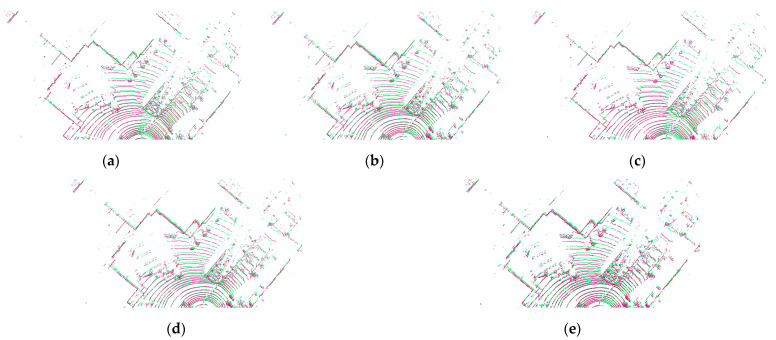
Visual analysis of different registration algorithms. (**a**) NDT, (**b**) NICP, (**c**) OURS, (**d**) TRICP, and (**e**) primitive point.

Figure 19 presents a visual analysis of four registration algorithms using data from real-vehicle experiments. The NDT, TRICP, and NICP registration algorithms show a low degree of coincidence, with significant differences in objects such as buildings and vehicles. On the other hand, after registration by the algorithm proposed in this paper, the degree of coincidence between point clouds is high, and the corresponding relationship is strong. Additionally, the proposed algorithm does not ignore any part of the point cloud, and the relationship between each point is preserved.

### 5.5. Metrological Characteristics Analysis of Lidar

The metrological characteristics of lidar include not only the measurement accuracy and angular resolution, but also other important factors, such as measurement range, point density, and reflectivity detection threshold. All of these characteristics can collectively affect the quality and accuracy of point cloud data collected by lidar, which can impact the result of point cloud registration. For example, the measurement accuracy of lidar determines its ability to accurately capture the 3D location of a target object. If the accuracy is low, the point cloud data collected by lidar will contain significant errors, which can negatively affect point cloud registration results. The point cloud data sources of the experiment are two different lidars, Pandar40P and Velodyne HDL-64E. Their metrological parameters are shown in Table 6.

It can be seen from Table 6 that Pandar40P has a longer measurement distance and a larger vertical field of view than Velodyne HDL-64E, but Velodyne HDL-64E has high measurement accuracy and high angular resolution. Table 7 shows the accuracy and time analysis of four different registration algorithms.

It can be seen from Table 7 that the algorithm proposed in this paper has a wide range of applicability and robustness, and can perform well on different types and specifications of lidar equipment. It can handle various noises and errors, and has high registration accuracy and reliability. At the same time, the algorithm can effectively overcome the differences between different lidars, and can also make an accurate registration of poor-quality or incomplete data.

## 6. Conclusions

The algorithm proposed in this paper is designed to address the challenges posed by the large scale, complex distribution, high noise, and strong sparsity of point cloud data collected by lidar during intelligent driving. The algorithm leverages regional feature extraction, mapping from local to global point clouds, and point cloud feature descriptors and local geometric features to achieve robust registration of point clouds for outdoor road scenes. The proposed algorithm has been verified through visualization effect analysis and experimental data analysis, demonstrating its high efficiency and accuracy in different working conditions and scenarios. The algorithm was also compared with three other point cloud registration algorithms under lidar with different metrological characteristics, and found to be robust and general. Finally, the algorithm has been validated on a real vehicle through point cloud registration experiments, showing its effectiveness and suitability for real-time applications. Overall, the algorithm proposed in this paper represents an important advancement in the field of point cloud registration and has significant potential for improving the efficiency and accuracy of intelligent driving systems.

Although the proposed algorithm has a more accurate registration performance, it still has some limitations. For example, the algorithm proposed in this paper only uses a single sensor to achieve point cloud registration. In our future work, it is intended to introduce a lightweight neural network that can fuse camera and lidar data to achieve precise estimation of lidar position and attitude. This will ensure the accuracy of point cloud registration even under extreme working conditions.

## Figures and Tables

**Table 1:** Registration results of Stanford University rabbit.

Algorithm	Root Mean Square Error/m	Time/s
NDT	0.032	29.32
TRICP	0.036	22.71
NICP	0.034	7.46
Ours	0.030	1.53

**Table 2:** Time comparison of algorithms.

Time/s									
Method	Scene1	Scene2	Scene3	Scene4	Scene5	Scene6	Scene7	Scene8	Scene9
NDT	13.80	12.50	12.75	15.47	18.48	14.83	19.42	16.08	14.32
TRICP	12.24	10.37	14.43	11.98	15.72	11.73	7.18	13.51	12.20
NICP	12.68	12.67	19.17	13.97	11.08	12.21	13.86	15.07	15.31
Ours	0.63	0.38	0.66	0.49	0.74	0.59	0.62	0.89	0.90

**Table 3:** Comparison of accuracy of algorithms.

Method	Road/m	Countryside/m	City/m
NDT	0.1427	0.1136	0.1241
TRICP	0.1346	0.08100	0.08766
NICP	0.1108	0.07548	0.08101
OURS	0.07231	0.06991	0.06766

**Table 4:** Lidar parameters.

Technical Parameter			
Principle of distance measurement	time-of-flight measurement	Scanning frequency	10 Hz, 20 Hz
Scanning principle	mechanical rotation	Vertical field of view	40° (−25~+15°)
Number of threads	40	Vertical angular resolution	minimum 0.33°
Detection distance	0.3~200 m	Horizontal field of view	360°
Measurement accuracy	±5 cm (0.3~0.5 m) ±2 cm (0.5~200 m)	Horizontal angular resolution	0.2° (10 Hz) 0.4° (20 Hz)

**Table 5:** Comparison of algorithm accuracy in real vehicle.

Algorithm	Root Mean Square Error/m
NDT	0.1229 ± 0.03925
NICP	0.1182 ± 0.04029
TRICP	0.1244 ± 0.06505
OURS	0.05996 ± 0.01751

**Table 6:** Metrological parameters of Pandar40P and Velodyne HDL-64E.

Device Model	Measuring Distance	Ranging Accuracy	Horizontal Field of View	Horizontal Angular Resolution	Vertical Field of View	Vertical Angular Resolution
Pandar40P	200 m	±2~±5 cm	360°	0.2~0.4°	40°	0.33~6°
Velodyne HDL_64E	120 m	±2 cm	360°	0.08~0.35°	26.9°	0.4°

**Table 7:** Accuracy and time analysis of four different registration algorithms.

Algorithm	Pandar40P		Velodyne HDL-64E	
	Root Square Mean Error/m	Time/s	Root Square Mean Error/m	Time/s
NDT	0.1229 ± 0.03925	27.8158 ± 6.2704	0.1169 ± 0.03418	23.5018 ± 6.4974
NICP	0.1182 ± 0.04029	21.1194 ± 9.9502	0.09416 ± 0.04526	17.4721 ± 9.9084
TRICP	0.1244 ± 0.06505	17.8066 ± 10.8586	0.1008 ± 0.05135	10.5351 ± 6.7416
OURS	0.05996 ± 0.01751	0.5739 ± 0.1227	0.06971 ± 0.01597	0.5873 ± 0.1470

## Data Availability

The data used to support the findings of this study are included in the article.
